# Dissecting the Causal Effects of Helicobacter pylori Serotypes on Gastric Cancer Risk: A Two-Sample Mendelian Randomization Study

**DOI:** 10.7759/cureus.89185

**Published:** 2025-07-31

**Authors:** Wentao Rao, Chenghong Xue, Donghui Gan, Binjian Liu

**Affiliations:** 1 Institute of Biomedicine and Biotechnology, Shenzhen Institutes of Advanced Technology, Chinese Academy of Sciences, Shenzhen, CHN; 2 Department of Clinical Medicine, Medcaptain Medical Technology Co. Ltd., Shenzhen, CHN

**Keywords:** causality, gastric cancer, genetic epidemiology, helicobacter pylori, mendelian randomization, virulence factor

## Abstract

Background

Although *Helicobacter* *pylori *infection is a primary risk factor for gastric cancer (GC), the specific bacterial components that causally drive carcinogenesis remain poorly understood. Traditional epidemiological studies are limited by confounding variables and the potential for reverse causation. This study aimed to dissect the causal effects of host antibody responses to various *H. pylori* antigens on GC risk using Mendelian randomization (MR).

Methodology

We conducted a two-sample MR study using summary statistics from large-scale genome-wide association studies (GWAS) of European-ancestry populations. Genetic instruments were selected for general *H. pylori* seropositivity and antibody levels against six antigens: cytotoxin-associated gene A (CagA), Catalase, GroEL, outer membrane protein (OMP), urease subunit A (UreA), and vacuolating cytotoxin A (VacA). The primary outcome was GC. Inverse-variance weighted (IVW) MR served as the main analysis, with comprehensive sensitivity analyses to assess the robustness of results. Multivariable MR (MVMR) was used to estimate the direct effects of each serotype, and a two-step mediation analysis was performed to explore potential mediating pathways.

Results

Genetically predicted general *H. pylori* seropositivity was causally associated with an increased risk of GC (odds ratio (OR): 1.12, 95% confidence interval (CI): 1.01-1.24; *P *= 0.027). The host antibody response to OMP showed a stronger causal effect (OR: 1.19, 95% CI: 1.08-1.30; *P *< 0.001). In contrast, no causal effects were observed for antibody responses to the classic virulence factors CagA or VacA (*P* > 0.05). In multivariable analysis, the effect of the anti-OMP response remained robust (OR: 1.18, 95% CI: 1.07-1.30; *P *= 0.001), while the association for general seropositivity was attenuated to null. Mediation analysis implicated tumor necrosis factor ligand superfamily member 18 (TNFSF18) as a potential mediator of the *H. pylori*-GC pathway, accounting for a substantial portion of the total effect (estimated at 47.0%), though this finding did not reach statistical significance (*P *= 0.077).

Conclusions

This MR study provides genetic evidence that the host immune response to *H. pylori* OMPs, rather than to classic virulence factors like CagA, is a key contributor to gastric carcinogenesis. This effect appears to be partially mediated by the inflammatory TNFSF18 pathway, suggesting that the chronic host-bacterial interactions at the gastric epithelial surface are critical to malignant transformation.

## Introduction

*Helicobacter pylori *infection represents one of the most significant modifiable risk factors for gastric cancer (GC), with the bacterium infecting approximately half of the global population and substantially increasing gastric adenocarcinoma risk [1,2]. Despite this well-established epidemiological association, the specific bacterial components that drive the progression from chronic infection to malignant transformation remain incompletely understood [3]. This knowledge gap has important implications for targeted prevention strategies, as not all *H. pylori-*infected individuals develop GC, and the heterogeneity in cancer risk may relate to differential exposure to specific bacterial virulence factors [3,4].

The complexity of *H. pylori* pathogenesis stems from the bacterium’s genetic diversity and arsenal of virulence factors that enable gastric colonization and immune manipulation. Key determinants include the cytotoxin-associated gene A (CagA) protein, vacuolating cytotoxin A (VacA), and surface proteins such as outer membrane protein (OMP), urease subunit A (UreA), heat shock protein GroEL, and catalase [4,5]. While past research has often focused on CagA-positive strains and their link to increased cancer risk, conventional epidemiological approaches face substantial limitations when investigating causal relationships between specific *H. pylori* antigens and GC [6].

Major methodological challenges include confounding by socioeconomic status, dietary habits, geographic location, and host genetic susceptibility, as well as concerns about reverse causation where gastric inflammation might alter immune responses to bacterial antigens [7]. These limitations have made it difficult to establish which bacterial components are causally responsible for carcinogenesis versus which are merely correlated markers of infection.

Mendelian randomization (MR) offers a powerful approach to overcome these obstacles by using the natural randomization that occurs during genetic inheritance [8]. MR uses genetic variants as instrumental variables-genetic markers that are associated with the exposure of interest but affect the outcome only through their influence on that exposure. This design mimics a randomized controlled trial because genetic variants are randomly allocated during meiosis, independent of environmental and lifestyle factors that typically confound observational studies.

To elucidate the causal relationship between *H. pylori* infection and GC risk, we designed a multi-faceted MR study. We performed two-sample MR to evaluate the causal effects of general *H. pylori* seropositivity and antibody responses against six key antigens, followed by multivariable MR (MVMR) to estimate the independent contribution of each serotype. Finally, we conducted a mediation analysis to explore potential biological pathways linking *H. pylori* infection to malignant transformation.

## Materials and methods

Study design and data sources

This study was designed and reported in accordance with the Strengthening the Reporting of Observational Studies in Epidemiology using Mendelian Randomization (STROBE-MR) statement [9]. We performed a two-sample MR analysis using publicly available summary statistics from large-scale genome-wide association studies (GWAS) [10-12]. This design leverages genetic variants associated with *H. pylori* serological responses as instrumental variables to investigate causal effects on GC risk while minimizing confounding and reverse causation. All datasets were derived from populations of European ancestry. An overview of the GWAS datasets is provided in Table 1. As this study used publicly available summary-level data, separate ethical approval was not required.

**Table 1:** Summary of GWAS datasets used in the study. GWAS, genome-wide association studies; SNP, single-nucleotide polymorphism; EBI, European Bioinformatics Institute

Dataset	GWAS ID	Data source	First author (Year)	Population	Sample size	No. SNPs
Anti-*Helicobacter pylori* IgG levels	ieu-b-4905	EBI	Chong (2021) [10]	European	4,683	12
*H. pylori* CagA antibody levels	ebi-a-GCST90006911	UK Biobank	Butler-Laporte (2020) [11]	European	985	14
*H. pylori* Catalase antibody levels	ebi-a-GCST90006912	UK Biobank	Butler-Laporte (2020) [11]	European	1,558	9
*H. pylori* GroEL antibody levels	ebi-a-GCST90006913	UK Biobank	Butler-Laporte (2020) [11]	European	2,716	4
*H. pylori* OMP antibody levels	ebi-a-GCST90006914	UK Biobank	Butler-Laporte (2020) [11]	European	2,640	9
*H. pylori* UreA antibody levels	ebi-a-GCST90006915	UK Biobank	Butler-Laporte (2020) [11]	European	2,251	10
*H. pylori* VacA antibody levels	ebi-a-GCST90006916	UK Biobank	Butler-Laporte (2020) [11]	European	1,571	15
Gastric cancer	ebi-a-GCST90018849	UK Biobank, FinnGen	Sakaue (2021) [12]	European	476,116	31

Instrumental variable selection and assumptions

For each exposure, single-nucleotide polymorphisms (SNPs) were selected as instrumental variables based on a genome-wide significance threshold (*P* < 5 × 10^-6^) [13]. To ensure independence, the selected SNPs were clumped using the 1,000 Genomes European reference panel, with a strict linkage disequilibrium threshold (*r*^2^ < 0.001) within a 10,000 kb window [13,14]. Palindromic SNPs with intermediate allele frequencies were excluded. The strength of the selected instruments was evaluated using the *F*-statistic, calculated as (β/se)^2^, where an *F*-statistic > 10 indicates sufficient strength against weak instrument bias [13,14].

**Figure 1:**
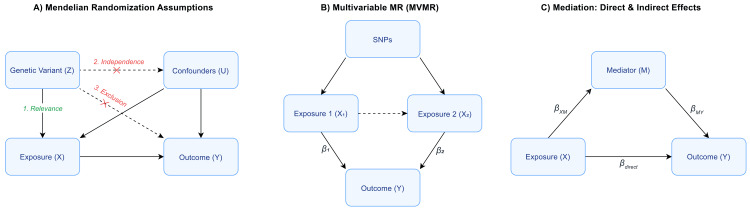
Conceptual frameworks for MR analyses. (A) The three core instrumental variable assumptions for a univariable MR study. (B) An MVMR model for estimating the independent causal effects of two exposures on an outcome. (C) A mediation model illustrating the decomposition of a total effect into its direct and indirect pathways. Image credit: Wentao Rao. MVMR, multivariable Mendelian randomization

MR analyses rely on three core assumptions (Figure 1A) [15]. First, the relevance assumption requires that the genetic variants are associated with *H. pylori* serological responses, which was ensured through the genome-wide significance threshold. Second, the independence assumption requires that genetic variants are not associated with confounders of the *H. pylori*-GC relationship, which was assessed by examining known pleiotropy and biological pathways. Third, the exclusion restriction assumption requires that genetic variants affect GC only through their effect on *H. pylori* immune responses, with no direct pathways or horizontal pleiotropy.

To satisfy the independence assumption, each selected SNP was queried against the GWAS ATLAS database to check for known associations with GC and potential confounders of the *H. pylori*-GC relationship, such as smoking, alcohol consumption, and dietary patterns. SNPs that were significantly (*P* < 1 × 10^−5^) associated with confounders in European participants were excluded [13,16]. The exclusion restriction assumption was supported by the MR-Egger intercept test and the MR-PRESSO (Pleiotropy RESidual Sum and Outlier) analysis. The clinical significance and related genes of the selected SNPs were acquired from the National Center for Biotechnology Information (NCBI) dbSNP (https://www.ncbi.nlm.nih.gov/snp/).

Primary analysis

The inverse-variance weighted (IVW) method served as the primary MR estimator, providing a weighted average of individual SNP effect estimates. This method assumes that all genetic variants are valid instruments and provides the most precise estimates when this assumption holds. Effect sizes were expressed as beta coefficients (β) and their corresponding odds ratios (OR = e^β^) per unit increase in log-odds of antibody seropositivity, with 95% confidence intervals calculated using standard errors from the IVW meta-analysis.

Sensitivity analysis

To assess the robustness of the findings and test for violations of the MR assumptions, several sensitivity analyses were performed. Results from weighted median and MR-Egger regression methods were also calculated, which provide more robust estimates under certain patterns of pleiotropy. Heterogeneity among SNPs was assessed using Cochran’s Q-statistic. Directional pleiotropy was evaluated using the MR-Egger intercept test. The MR-PRESSO test was used to identify and correct for potential pleiotropic outliers [17]. A Mixture of Experts (MR-MOE) approach was used in addition to choose the most appropriate method amongst several MR tests using a machine learning algorithm [18]. Finally, a leave-one-out analysis was conducted to ensure that the causal estimate was not driven by any single influential SNP.

Bidirectional and multivariable MR

Bidirectional MR was performed to test for evidence of reverse causation (i.e., that a predisposition to GC might influence *H. pylori* serostatus). MVMR was conducted to estimate the direct causal effect of each serotype on GC, mutually adjusting for the genetic effects of the other serotypes (Figure 1B) [19]. This analysis is critical for identifying the primary causal antigen(s) from a set of correlated exposures.

Mediation analysis

To explore potential biological mechanisms, a two-step mediation analysis was conducted (Figure 1C) [19,20]. First, the causal effect of the significant *H. pylori* exposure(s) on potential inflammatory mediators (e.g., C-reactive protein (CRP), interleukins (IL), tumor necrosis factors (TNFs)) was estimated as β_XM_. Then, MVMR was used to estimate the direct effect of the mediator on GC β_MY_ after adjusting for the exposure(s). Finally, the indirect effect of exposure(s) on GC β_indirect_ was calculated as: $&beta;_{indirect}=&beta;_{XM}\cdot &beta;_{MY}$.

The mediation analysis estimated both direct effects (*H. pylori* → GC) and indirect effects (*H. pylori* → mediator → GC), with the proportion mediated calculated as the ratio of indirect to total effects ($&beta;_{indirect}/&beta;_{total}$). Statistical significance of mediation was assessed using the delta method to calculate standard errors and confidence intervals for indirect effects [21].

Statistical and data visualization software

All analyses were conducted in R (version 4.4.3) using the TwoSampleMR package [14]. Data visualization and plotting were performed using the matplotlib library in Python (version 3.12).

## Results

Univariable causal effects of *H. pylori* serotypes on GC

After selection and quality control, independent and strong genetic instruments for all seven *H. pylori*-related exposures were obtained (all F-statistics > 10). No SNPs exhibited strong associations with GC or potential confounding traits, and dbSNP searches revealed no clinical significance annotations for any of the selected variants. The full lists of SNPs were provided in Tables 2-8.

**Table 2:** Lists of genetic instruments used for general seropositivity (ieu-b-4905). Summary statistics were obtained from genome-wide association studies (GWAS). Associations were tested using linear regression for continuous traits or logistic regression for binary traits. The beta coefficient represents the change in trait level or the log odds ratio per effect allele. F-statistics were calculated as (β/se)^2^. SNP, single-nucleotide polymorphism; CHR, chromosome; POS, position; EAF, effect allele frequency; SE, standard error; NA, not available

SNP	CHR	POS	Effect/Other allele	EAF	Beta	SE	*P*-value	F-statistics	Gene
rs41263973	1	32162810	A/G	0.034	0.316	0.067	2.74E-06	22.1	COL16A1
rs2169557	2	20244954	T/C	0.488	-0.106	0.023	4.55E-06	21.1	LAPTM4A
rs77516628	4	62125618	T/A	0.089	0.188	0.040	2.94E-06	21.9	ADGRL3; LOC124900173
rs72708546	4	170153219	A/G	0.059	-0.230	0.048	1.90E-06	22.8	SH3RF1
rs35030589	6	32672903	A/G	0.132	-0.175	0.034	3.40E-07	26.1	NA
rs117912702	6	166062930	A/G	0.020	0.405	0.087	3.02E-06	21.9	PDE10A
rs73512476	11	86585900	T/G	0.077	0.213	0.044	1.47E-06	23.3	PRSS23
rs17502937	13	30440740	T/G	0.020	-0.397	0.085	2.89E-06	22.0	NA
rs74045808	14	34457396	T/C	0.108	-0.175	0.038	4.49E-06	21.1	LOC102724945
rs78825412	15	83512621	A/C	0.032	0.318	0.069	3.64E-06	21.5	HOMER2
rs12591869	15	96674267	A/C	0.270	-0.129	0.027	1.29E-06	23.5	NR2F2-AS1; LOC112268156
rs55871438	15	75411120	C/T	0.039	0.299	0.065	4.02E-06	21.3	NA

**Table 3:** Lists of genetic instruments used for anti-CagA IgG (ebi-a-GCST90006911). Summary statistics were obtained from genome-wide association studies (GWAS). Associations were tested using linear regression for continuous traits or logistic regression for binary traits. The beta coefficient represents the change in trait level or the log odds ratio per effect allele. F-statistics were calculated as (β/se)^2^. SNP, single-nucleotide polymorphism; CHR, chromosome; POS, position; EAF, effect allele frequency; SE, standard error; NA, not available

SNP	CHR	POS	Effect/Other allele	EAF	Beta	SE	*P*-value	F-statistics	Gene
rs116421363	1	181820561	C/A	0.363	-0.223	0.048	2.68E-06	22.0	NA
rs75170215	3	25908239	C/T	0.021	0.743	0.156	2.02E-06	22.6	LINC00692
rs75740599	4	159123611	A/G	0.253	0.236	0.051	4.27E-06	21.1	GASK1B-AS1
rs3998182	6	32561915	C/T	0.276	0.285	0.055	2.04E-07	27.0	NA
rs571061419	6	162760485	A/G	0.074	-0.392	0.085	4.21E-06	21.2	PRKN
rs6530847	8	15111565	A/T	0.391	-0.210	0.045	3.41E-06	21.6	NA
rs4268452	10	81319354	T/C	0.071	-0.385	0.084	4.62E-06	21.0	SFTPA2
rs117827497	11	105212623	G/A	0.030	-0.584	0.125	3.10E-06	21.8	LOC105369468
rs138363822	11	1606147	G/A	0.426	0.218	0.048	4.30E-06	21.1	KRTAP5-1; KRTAP5-AS1
rs117537486	11	94351805	C/G	0.031	0.597	0.130	4.22E-06	21.2	PIWIL4; PIWIL4-AS1
rs149747348	12	98890443	C/G	0.013	-0.895	0.194	3.92E-06	21.3	LINC02453
rs56264437	13	102123887	A/G	0.604	0.234	0.049	1.99E-06	22.6	ITGBL1
rs11858369	15	90495509	G/A	0.068	0.438	0.086	4.18E-07	25.6	NA
rs118006294	16	10974221	C/T	0.050	-0.475	0.102	3.04E-06	21.8	CIITA

**Table 4:** Lists of genetic instruments used for anti-Catalase IgG (ebi-a-GCST90006912). Summary statistics were obtained from genome-wide association studies (GWAS). Associations were tested using linear regression for continuous traits or logistic regression for binary traits. The beta coefficient represents the change in trait level or the log odds ratio per effect allele. F-statistics were calculated as (β/se)^2^. SNP, single-nucleotide polymorphism; CHR, chromosome; POS, position; EAF, effect allele frequency; SE, standard error; NA, not available

SNP	CHR	POS	Effect/Other allele	EAF	Beta	SE	*P*-value	F-statistics	Gene
rs190890483	2	157083346	A/G	0.012	-0.772	0.157	8.63E-07	24.2	LINC01876
rs72808426	2	58133048	T/C	0.035	-0.434	0.094	3.79E-06	21.4	VRK2
rs58679787	2	16452055	T/C	0.011	0.777	0.168	3.84E-06	21.3	NA
rs343686	6	80607062	C/A	0.621	-0.175	0.037	1.75E-06	22.9	NA
rs6456714	6	26335346	G/C	0.632	0.171	0.036	2.18E-06	22.4	LOC124901288
rs77162569	7	148264987	T/C	0.025	-0.556	0.109	3.27E-07	26.1	LOC124901766
rs17647677	13	46749781	C/T	0.041	-0.394	0.085	3.65E-06	21.4	LCP1
rs927062	14	33095049	G/A	0.214	-0.198	0.042	2.60E-06	22.1	AKAP6
rs147016571	16	25837192	G/A	0.010	-0.840	0.176	1.76E-06	22.8	HS3ST4

**Table 5:** Lists of genetic instruments used for anti-GroEL IgG (ebi-a-GCST90006913). Summary statistics were obtained from genome-wide association studies (GWAS). Associations were tested using linear regression for continuous traits or logistic regression for binary traits. The beta coefficient represents the change in trait level or the log odds ratio per effect allele. F-statistics were calculated as (β/se)^2^. SNP, single-nucleotide polymorphism; CHR, chromosome; POS, position; EAF, effect allele frequency; SE, standard error; NA, not available

SNP	CHR	POS	Effect/Other allele	EAF	Beta	SE	*P*-value	*F*-statistics	Gene
rs3104037	6	107139657	T/C	0.614	0.134	0.028	1.90E-06	22.7	NA
rs61680606	10	128939945	C/A	0.226	0.152	0.032	2.38E-06	22.3	DOCK1; INSYN2A
rs117341694	13	66889675	A/G	0.013	0.543	0.116	2.73E-06	22.0	PCDH9
rs1367344	18	71479072	C/T	0.224	-0.155	0.033	2.68E-06	22.0	NA

**Table 6:** Lists of genetic instruments used for anti-OMP IgG (ebi-a-GCST90006914). Summary statistics were obtained from genome-wide association studies (GWAS). Associations were tested using linear regression for continuous traits or logistic regression for binary traits. The beta coefficient represents the change in trait level or the log odds ratio per effect allele. F-statistics were calculated as (β/se)^2^. SNP, single-nucleotide polymorphism; CHR, chromosome; POS, position; EAF, effect allele frequency; SE, standard error; NA, not available

SNP	CHR	POS	Effect/Other allele	EAF	Beta	SE	*P*-value	F-statistics	Gene
rs703135	1	158678095	A/G	0.418	-0.133	0.028	2.09E-06	22.5	NA
rs55862931	2	2672878	C/G	0.258	-0.146	0.032	3.82E-06	21.4	NA
rs143477841	2	24042805	A/G	0.012	-0.573	0.125	4.37E-06	21.1	ATAD2B
rs9276733	6	32766026	G/A	0.230	0.196	0.036	8.14E-08	28.8	NA
rs3104361	6	32652278	C/T	0.582	0.181	0.029	6.48E-10	38.2	NA
rs60949128	8	41936404	A/C	0.064	-0.277	0.056	8.53E-07	24.2	KAT6A-AS1
rs116944686	8	101374808	A/G	0.023	0.418	0.091	4.78E-06	20.9	LOC124901991
rs8019346	14	65353011	A/G	0.085	0.229	0.050	4.24E-06	21.2	LOC105370534
rs1892331	20	59326926	G/A	0.032	0.377	0.076	7.40E-07	24.5	LOC105372699

**Table 7:** Lists of genetic instruments used for anti-UreA IgG (ebi-a-GCST90006915) Summary statistics were obtained from genome-wide association studies (GWAS). Associations were tested using linear regression for continuous traits or logistic regression for binary traits. The beta coefficient represents the change in trait level or the log odds ratio per effect allele. F-statistics were calculated as (β/se)^2^. SNP, single-nucleotide polymorphism; CHR, chromosome; POS, position; EAF, effect allele frequency; SE, standard error; NA, not available

SNP	CHR	POS	Effect/Other allele	EAF	Beta	SE	*P*-value	F-statistics	Gene
rs9289888	3	152849257	T/C	0.066	0.272	0.057	2.12E-06	22.5	NA
rs4689912	4	4659633	C/T	0.267	-0.158	0.033	1.90E-06	22.7	STX18-AS1; LOC124900165
rs71569678	6	23380648	C/A	0.060	0.340	0.061	2.99E-08	30.7	LOC105374976; LOC124900213
rs75477465	8	68794331	A/G	0.034	0.375	0.080	3.03E-06	21.8	NA
rs61907106	12	4003258	A/G	0.048	-0.359	0.070	3.27E-07	26.1	PARP11-AS1
rs12820262	12	129244638	A/G	0.177	0.206	0.039	1.59E-07	27.5	NA
rs60386004	14	80136052	T/A	0.158	0.192	0.041	2.49E-06	22.2	NRXN3
rs143570118	17	76370080	T/C	0.029	0.434	0.093	2.76E-06	22.0	SOCS3-DT
rs117994655	19	8188056	A/G	0.033	0.402	0.083	1.21E-06	23.6	FBN3
rs134748	22	26543287	T/G	0.653	-0.142	0.031	3.50E-06	21.5	LOC102724801

**Table 8:** Lists of genetic instruments used for anti-VacA IgG (ebi-a-GCST90006916). Summary statistics were obtained from genome-wide association studies (GWAS). Associations were tested using linear regression for continuous traits or logistic regression for binary traits. The beta coefficient represents the change in trait level or the log odds ratio per effect allele. F-statistics were calculated as (β/se)^2^. SNP, single-nucleotide polymorphism; CHR, chromosome; POS, position; EAF, effect allele frequency; SE, standard error; NA, not available

SNP	CHR	POS	Effect/Other allele	EAF	Beta	SE	*P*-value	F-statistics	Gene
rs113063793	1	217309978	T/A	0.056	0.373	0.076	7.97E-07	24.4	ESRRG
rs72645538	1	9453174	G/A	0.012	0.753	0.164	4.66E-06	21.0	NA
rs113845906	3	94372336	A/G	0.045	0.413	0.085	9.97E-07	23.9	LOC105373992
rs1530121	5	124624902	C/T	0.863	-0.266	0.051	1.96E-07	27.1	LOC101927421
rs372744619	6	32434141	G/A	0.011	0.887	0.174	3.29E-07	26.1	NA
rs77497849	7	32049959	G/A	0.119	0.264	0.054	9.13E-07	24.1	PDE1C
rs10246445	7	2981123	C/A	0.028	-0.488	0.107	4.92E-06	20.9	CARD11
rs7019543	9	110365310	T/G	0.320	0.181	0.038	2.01E-06	22.6	LOC105376205
rs59104649	10	129150690	T/A	0.014	0.683	0.147	3.44E-06	21.6	DOCK1
rs117077218	10	90083333	T/C	0.017	0.640	0.139	3.95E-06	21.3	RNLS; LOC101929727
rs148556020	12	28904914	A/T	0.017	0.631	0.137	4.44E-06	21.1	LOC105369711
rs11044935	12	20062085	T/A	0.039	0.428	0.091	2.22E-06	22.4	NA
rs73500239	16	1332341	C/G	0.145	0.236	0.050	2.38E-06	22.3	NA
rs9606224	22	20034187	T/C	0.030	0.507	0.102	6.91E-07	24.6	TANGO2
rs133537	22	48595971	C/T	0.615	-0.174	0.037	2.25E-06	22.4	NA

**Figure 2:**
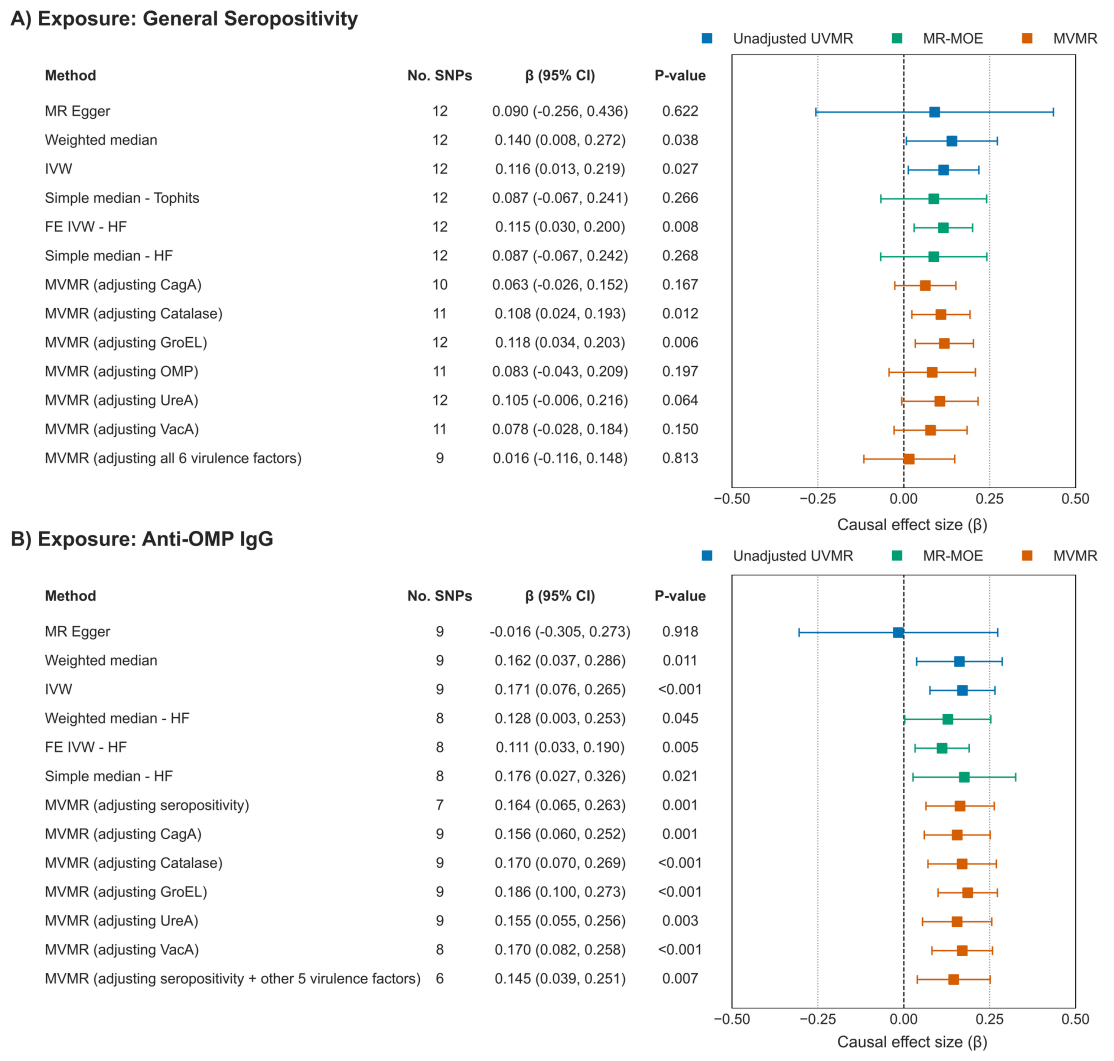
Forest plot of MR estimates for the causal effect of H. pylori exposures on GC. The plot shows estimates from univariable, MR-MOE, and MVMR methods for (A) general seropositivity and (B) anti-OMP IgG. MR, Mendelian randomization; SNP, single nucleotide polymorphism; CI, confidence interval; IVW, inverse-variance weighted; FE, fixed effect; HF, heterogeneity filtering; MVMR, multivariable Mendelian randomization; MOE, Mixture of Experts; GC, gastric cancer

**Figure 3:**
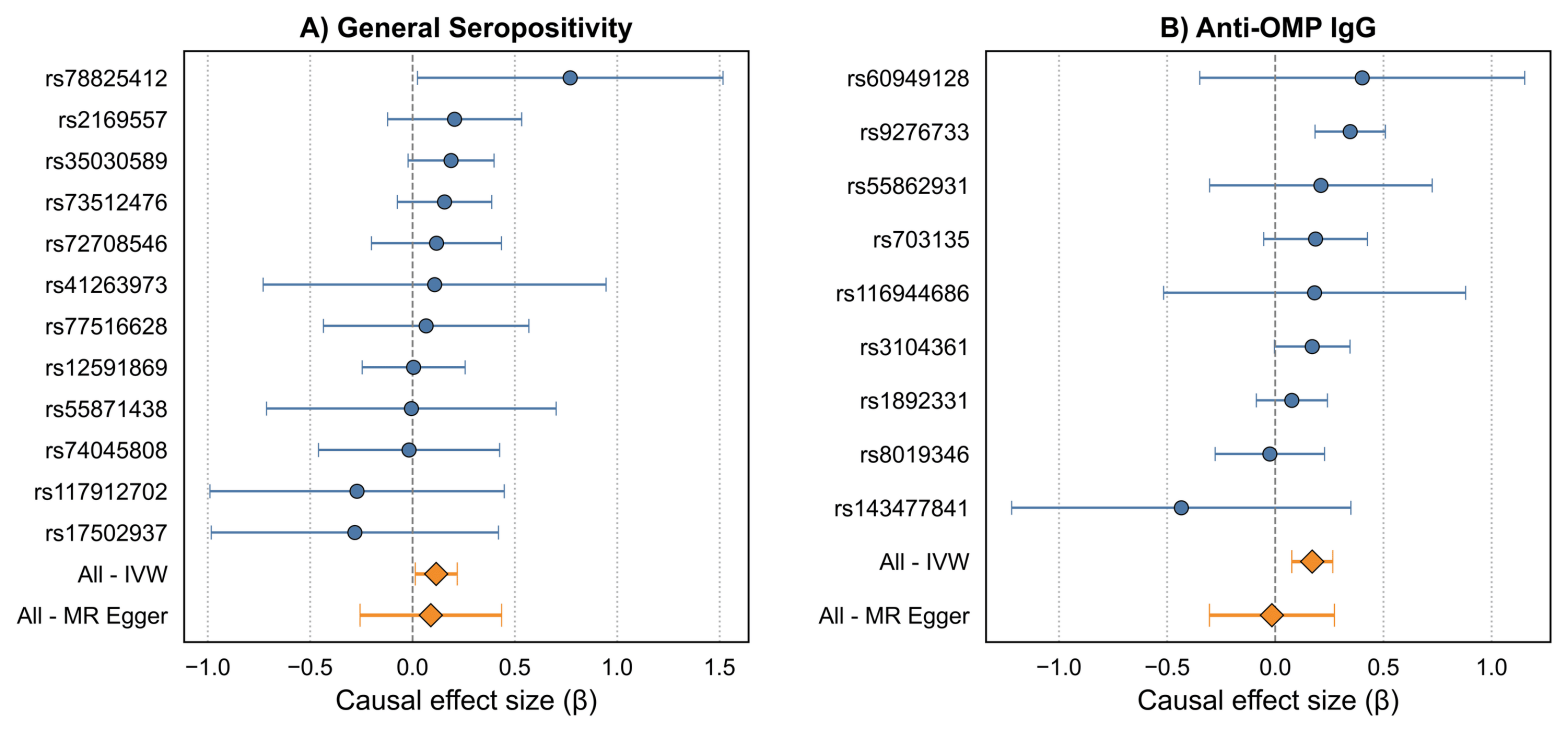
Forest plot of single-SNP causal estimates for the effect of H. pylori exposures on GC. Each point represents the causal estimate derived from a single genetic variant for (A) general seropositivity and (B) anti-OMP IgG. MR, Mendelian randomization; GC, gastric cancer; SNP, single nucleotide polymorphism; IVW, inverse-variance weighted

**Figure 4:**
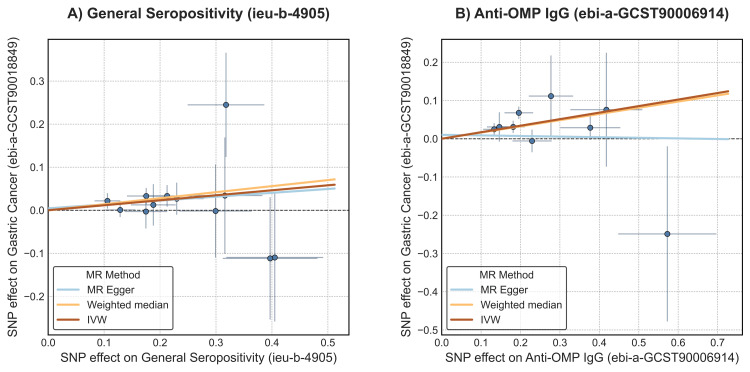
Scatter plots of SNP-exposure versus SNP-outcome effects. (A) General seropositivity, (B) anti-OMP IgG. MR, Mendelian randomization; SNP, single-nucleotide polymorphism; IVW, inverse-variance weighted

The primary UVMR analysis revealed statistically significant causal effects for two exposures on the risk of GC (Table 9). Genetically predicted general *H. pylori* seropositivity was associated with a 12% increase in GC risk (IVW β: 0.116, 95% CI: 0.013-0.219; OR: 1.12; 95% CI: 1.01-1.24; *P *= 0.027). A stronger effect was observed for antibody response to OMP, which was associated with a 19% increase in GC risk (IVW β: 0.171, 95% CI: 0.076-0.265; OR: 1.19; 95% CI: 1.08-1.30; *P *< 0.001). The results were consistent across multiple analyses, including the weighted median and MR-MOE methods (Figure 2). Forest plots of single-SNP causal estimates for the effect were presented in Figure 3. Scatter plots for the main analyses of general *H. pylori* seropositivity and anti-OMP IgG are shown in Figure 4. While the primary IVW and weighted median methods showed a significant positive association between anti-OMP IgG and GC, the MR-Egger estimate had a negative direction, though it was not statistically significant (β = -0.016, *P *= 0.918). This divergence can occur when there is potential for residual pleiotropy. Although the MR-Egger intercept test did not indicate significant directional pleiotropy (*P *= 0.225), the consistency of the positive effect across the more powerful IVW and weighted median analyses provides confidence in the primary finding.

**Table 9:** Univariable MR estimates for the causal effect of H. pylori serotypes on GC risk. MR, Mendelian randomization; GC, gastric cancer; SNP, single-nucleotide polymorphism; CI, confidence interval; OR, odds ratio; IVW, inverse-variance weighted

Exposure	Method	No. of SNPs	β (95% CI)	OR (95% CI)	*P*-value
General seropositivity	MR-Egger	12	0.090 (-0.256-0.436)	1.09 (0.77-1.55)	0.622
	Weighted median	12	0.140 (0.008-0.272)	1.15 (1.01-1.31)	0.038
	IVW	12	0.116 (0.013-0.219)	1.12 (1.01-1.24)	0.027
Anti-CagA IgG	MR-Egger	14	0.073 (-0.108-0.253)	1.08 (0.90-1.29)	0.446
	Weighted median	14	0.013 (-0.087-0.113)	1.01 (0.92-1.12)	0.799
	IVW	14	-0.009 (-0.078-0.061)	0.99 (0.92-1.06)	0.806
Anti-Catalase IgG	MR-Egger	9	0.083 (-0.213-0.379)	1.09 (0.81-1.46)	0.600
	Weighted median	9	0.012 (-0.118-0.142)	1.01 (0.89-1.15)	0.856
	IVW	9	0.022 (-0.084-0.129)	1.02 (0.92-1.14)	0.685
Anti-GroEL IgG	MR-Egger	4	0.123 (-0.517-0.764)	1.13 (0.60-2.15)	0.742
	Weighted median	4	-0.059 (-0.270-0.151)	0.94 (0.76-1.16)	0.582
	IVW	4	-0.050 (-0.225-0.125)	0.95 (0.80-1.13)	0.574
Anti-OMP IgG	MR-Egger	9	-0.016 (-0.305-0.273)	0.98 (0.74-1.31)	0.918
	Weighted median	9	0.162 (0.037-0.286)	1.18 (1.04-1.33)	0.011
	IVW	9	0.171 (0.076-0.265)	1.19 (1.08-1.30)	<0.001
Anti-UreA IgG	MR-Egger	10	0.090 (-0.230-0.410)	1.09 (0.79-1.51)	0.597
	Weighted median	10	0.024 (-0.073-0.121)	1.02 (0.93-1.13)	0.624
	IVW	10	0.002 (-0.090-0.094)	1.00 (0.91-1.10)	0.966
Anti-VacA IgG	MR-Egger	15	0.083 (-0.026-0.191)	1.09 (0.97-1.21)	0.161
	Weighted median	15	0.071 (-0.018-0.160)	1.07 (0.98-1.17)	0.120
	IVW	15	0.035 (-0.024-0.093)	1.04 (0.98-1.10)	0.242

In contrast, no evidence of a causal effect for antibody levels against the classic virulence factors CagA, VacA, or other tested antigens (Catalase, GroEL, UreA) on GC risk was found (all *P* > 0.05).

Sensitivity and bidirectional analyses

**Figure 5:**
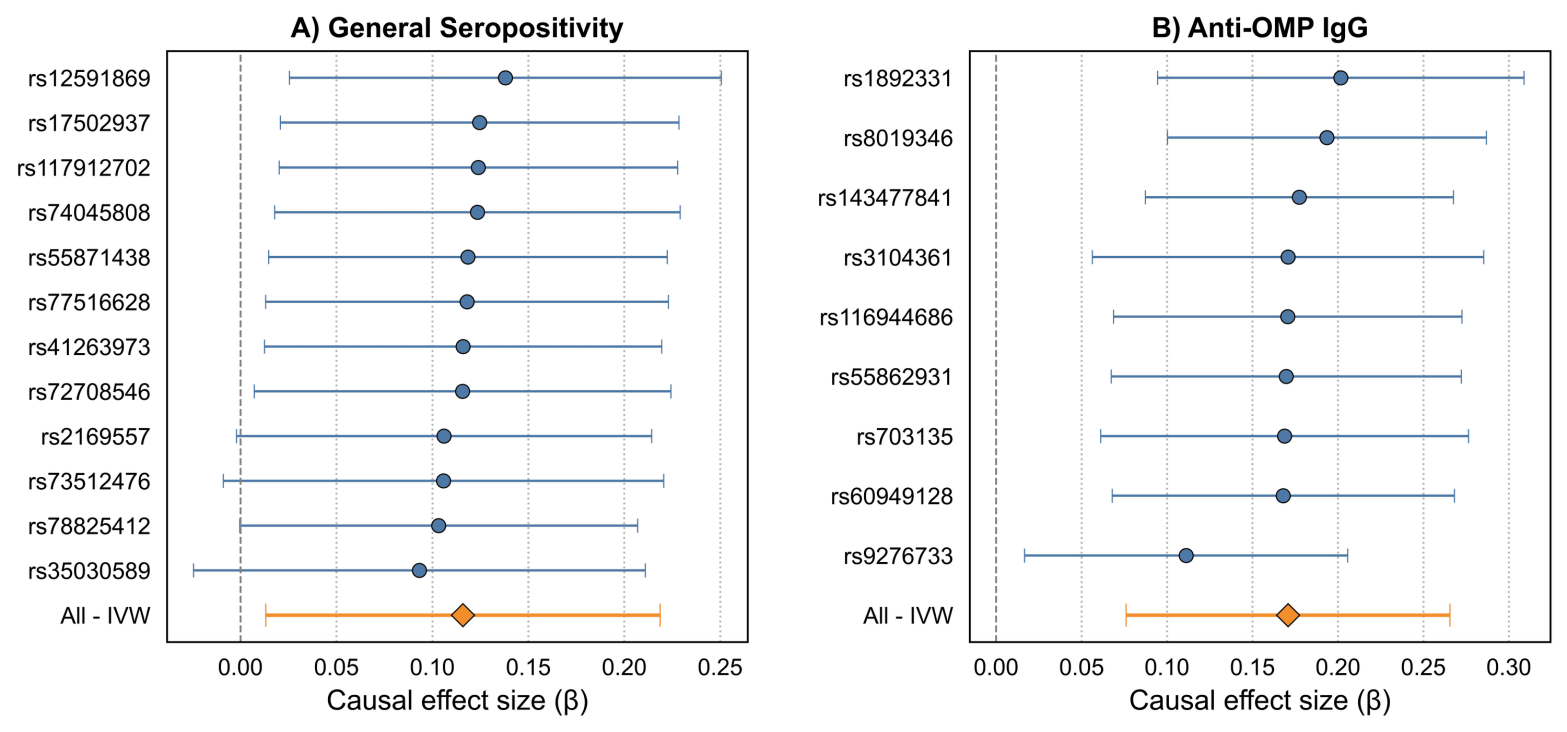
Leave-one-out analysis plots. Each point represents the causal estimate after removing a single genetic variant for (A) general seropositivity and (B) anti-OMP IgG. IVW, inverse-variance weighted; OMP, outer membrane protein

Comprehensive sensitivity analyses supported the validity of the main findings. No evidence of significant heterogeneity was found between instruments in the IVW analyses for general seropositivity or anti-OMP IgG (Cochran’s Q *P* > 0.05 for both; Table 10). The MR-Egger intercept test showed no evidence of directional pleiotropy for any of the exposures (all *P* > 0.05; Table 11). No outliers were identified by MR-PRESSO. Furthermore, leave-one-out analyses demonstrated that the causal estimates were not driven by any single SNP (Figure 5). Bidirectional MR found no evidence of a causal effect of GC liability on any of the *H. pylori* serostatus phenotypes (data not shown).

**Table 10:** Heterogeneity test results. Heterogeneity was assessed using Cochran’s Q test. A *P*-value > 0.05 indicates no significant heterogeneity. MR, Mendelian randomization; IVW, inverse-variance weighted; *Q*, Cochran’s *Q* statistic; DF, degrees of freedom; OMP, outer membrane protein; UreA, urease subunit A; VacA, vacuolating cytotoxin A

Exposure	Method	Q	DF	*P*-value
General seropositivity	MR Egger	7.38	10	0.689
	IVW	7.40	11	0.766
Anti-CagA IgG	MR-Egger	8.59	12	0.737
	IVW	9.51	13	0.734
Anti-Catalase IgG	MR-Egger	3.57	7	0.828
	IVW	3.76	8	0.878
Anti-GroEL IgG	MR-Egger	1.79	2	0.408
	IVW	2.09	3	0.553
Anti-OMP IgG	MR-Egger	8.58	7	0.284
	IVW	10.76	8	0.216
Anti-UreA IgG	MR-Egger	14.78	8	0.064
	IVW	15.37	9	0.081
Anti-VacA IgG	MR-Egger	11.64	13	0.557
	IVW	12.68	14	0.552

**Table 11:** Pleiotropy test results. Directional pleiotropy was assessed using the MR-Egger intercept test. A *P*-value > 0.05 suggests no significant directional pleiotropy. SE, standard error; MR, Mendelian randomization; OMP, outer membrane protein; UreA, urease subunit A; VacA, vacuolating cytotoxin A

Exposure	Egger intercept	SE	*P*-value
General seropositivity	0.0045	0.0291	0.880
Anti-CagA IgG	-0.0266	0.0278	0.358
Anti-Catalase IgG	-0.0138	0.0319	0.678
Anti-GroEL IgG	-0.0269	0.0488	0.637
Anti-OMP IgG	0.0393	0.0295	0.225
Anti-UreA IgG	-0.0197	0.0350	0.588
Anti-VacA IgG	-0.0232	0.0228	0.327

Multivariable MR

To disentangle the effects of the correlated *H. pylori* exposures, MVMR was performed. When adjusting for general seropositivity, the effect of anti-OMP IgG on GC remained robust and significant (MVMR β: 0.164, 95% CI: 0.065-0.263; OR: 1.18; 95% CI: 1.07-1.30; *P *= 0.001). Conversely, when adjusting for anti-OMP IgG, the effect of general seropositivity was attenuated to the null (MVMR β: 0.083, 95% CI: -0.043 to 0.209; OR: 1.09; 95% CI: 0.96-1.23; *P *= 0.197).

In a final comprehensive model including general seropositivity and all six virulence factors, the host antibody response to OMP was the only exposure that retained a significant causal association with GC (MVMR β: 0.145, 95% CI: 0.039-0.251; OR: 1.16; 95% CI: 1.04-1.29; *P *= 0.007). The effect of general seropositivity was fully attenuated (MVMR β: 0.016, 95% CI: -0.116 to 0.148; OR: 1.02; 95% CI: 0.89-1.16; *P *= 0.813) (Table 12). These results suggest that the immune response to outer membrane proteins is the primary contributor to the observed association between *H. pylori* serostatus and GC.

**Table 12:** Multivariable MR estimates for the direct causal effects of H. pylori serotypes on GC. MR, Mendelian randomization; GC, gastric cancer; SNP, single-nucleotide polymorphism; OR, odds ratio; CI, confidence interval

Exposure	No. of SNPs	β (95% CI)	OR (95% CI)	*P*-value
General seropositivity	9	0.016 (-0.116 to 0.148)	1.02 (0.89-1.16)	0.813
Anti-CagA IgG	6	-0.016 (-0.101 to 0.070)	0.98 (0.90-1.07)	0.721
Anti-Catalase IgG	6	-0.019 (-0.135 to 0.097)	0.98 (0.87-1.10)	0.747
Anti-GroEL IgG	2	0.047 (-0.207 to 0.301)	1.05 (0.81-1.35)	0.718
Anti-OMP IgG	6	0.145 (0.039-0.251)	1.16 (1.04-1.29)	0.007
Anti-UreA IgG	10	0.027 (-0.064 to 0.119)	1.03 (0.94-1.13)	0.560
Anti-VacA IgG	12	-0.015 (-0.142 to 0.112)	0.99 (0.87-1.12)	0.817

Mediation analysis

Several inflammatory biomarkers were explored as potential mediators of the *H. pylori*-GC pathway. While no evidence was found for mediation by CRP or major ILs, our analysis identified tumor necrosis factor ligand superfamily member 18 (TNFSF18, also known as GITRL) as a potential mediator of the effect of general *H. pylori* seropositivity on GC. The indirect effect via TNFSF18 was at the margin of statistical significance (β_indirect_: 0.054, 95% CI: -0.006 to 0.115; OR: 1.06, 95% CI: 0.99-1.12; *P *= 0.077) and was estimated to account for 47.0% of the total causal effect, indicating a potential role for the GITR-GITRL signaling pathway in *H. pylori*-driven carcinogenesis. The mediation pathway consisted of a negative association between *H. pylori* seropositivity and TNFSF18 levels (β: -0.475; *P *= 0.075) and a strong negative association between TNFSF18 and GC risk (β: -0.115; *P* = 1.09 × 10^-64^). This suggests that *H. pylori* infection may increase GC risk partially through suppression of TNFSF18-mediated immune responses.

## Discussion

In this comprehensive, two-sample MR study, we dissected the causal relationships between various *H. pylori* antibody responses and the risk of GC. Our primary finding is that while general *H. pylori* seropositivity is causally linked to GC, the host immune response to bacterial OMPs appears to be a key, independent contributor to this effect. Notably, we found no genetic evidence to support a causal role for antibody responses to the classic virulence factors CagA or VacA in this European-ancestry population, challenging the long-held paradigm that these specific toxins are the principal drivers of oncogenesis.

The identification of the anti-OMP response as a key contributor is a significant finding. OMPs, such as the well-characterized adhesins BabA and SabA, are critical for bacterial survival, adhesion to gastric epithelial cells, and interaction with the host immune system [22,23]. Unlike intracellular or secreted virulence factors, OMPs are constitutively expressed on the bacterial surface and are therefore continuously exposed to host immune surveillance throughout the infection process. A robust and chronic immune response to these surface proteins likely reflects a high bacterial load and persistent, direct interaction with the gastric mucosa, which are prerequisites for the chronic inflammation that underlies the Correa’s cascade [24,25]. Our multivariable analysis, which showed the attenuation of the general seropositivity effect after accounting for anti-OMP IgG, suggests that the *general* risk is largely a proxy for the more specific and pathologically critical response to these surface antigens.

Mechanistically, the role of OMPs extends far beyond simple adhesion. This initial binding is an active, critical step that enables the delivery of the bacterium’s most potent virulence factors, such as the CagA oncoprotein, via the Type IV Secretion System (T4SS) [26]. The interaction between the adhesin HopQ and host Carcinoembryonic Antigen-Related Cell Adhesion Molecules (CEACAMs) is now understood to be indispensable for efficient CagA translocation [23,27]. Similarly, BabA-mediated adherence potentiates T4SS function, thereby amplifying downstream inflammatory signaling and cellular transformation driven by CagA [23]. This synergistic relationship, where OMPs act as gatekeepers for the oncogenic activity of other toxins, underscores their central role in initiating the carcinogenic cascade.

Furthermore, OMPs function as direct instigators of pro-tumorigenic signaling. The outer inflammatory protein A (OipA), for instance, can trigger the activation of key oncogenic pathways, including the PI3K/Akt and MAPK/Erk cascades, which promote cell proliferation and survival. Concurrently, OMP engagement with host receptors, particularly the HopQ-CEACAM interaction, is a potent activator of the NF-κB pathway, driving the expression of pro-inflammatory cytokines like IL-8 and establishing a chronic inflammatory microenvironment [23]. This sustained inflammation is a major source of reactive oxygen species (ROS) that cause host DNA damage, a process further exacerbated by the direct activity of OMPs like γ-glutamyl transpeptidase (GGT) [28,29]. This multi-faceted assault-combining the potentiation of other toxins with the direct activation of oncogenic signaling and the fueling of a mutagenic inflammatory state-provides a robust mechanistic explanation for our finding that a strong, specific immune response to OMPs is causally linked to GC development.

Our exploratory mediation analysis points towards a novel and mechanistically plausible pathway that may explain a significant portion of *H. pylori*’s carcinogenic effect: the suppression of the GITR-GITRL immune checkpoint pathway. The results suggest a compelling two-part mechanism: first, genetically predicted *H. pylori* infection causally leads to a downregulation of circulating TNFSF18 (GITRL); second, genetically predicted higher levels of TNFSF18 are independently and causally associated with a lower risk of GC. Taken together, this implies that *H. pylori* may promote carcinogenesis by actively suppressing a protective immune pathway. The GITR-GITRL system is a multifaceted regulator of immune homeostasis, critical for the co-stimulation and survival of both effector and regulatory T-cells [30]. Chronic downregulation of TNFSF18 by *H. pylori* could dysregulate this balance, potentially leading to T-cell exhaustion and a failure of immune surveillance against transformed gastric epithelial cells. This creates a more nuanced picture than simple pro-inflammation; instead, it suggests *H. pylori* fosters a specific type of ineffective chronic inflammation, allowing it to persist while simultaneously impairing the host’s ability to eliminate nascent cancer cells. Although the statistical evidence for this mediation pathway was at the margin of significance, its biological coherence and the substantial proportion of the total effect it potentially explains (47%) make it a compelling, hypothesis-generating finding that warrants further experimental investigation.

This study’s null finding for classic *H. pylori* virulence factors, particularly CagA and VacA, must be interpreted with caution. In MR, where gene-exposure associations can be weak, focusing solely on statistical significance may lead to type II errors [8]. The lack of a statistically significant association does not constitute definitive evidence for the absence of a causal effect. A plausible explanation is that our analysis was underpowered to detect a modest effect. The GWAS for the specific virulence factor antibody responses had substantially smaller sample sizes (*N* < 3,000) compared to the outcome GWAS (*N* > 476,000). This can result in genetic instruments that, while meeting the minimum F-statistic threshold, explain only a very small proportion of the variance in the exposure, leading to wide confidence intervals and an inability to resolve a true, but modest, causal relationship. Alternatively, it is conceivable that in these European-ancestry cohorts, the population-level oncogenic impact of these specific toxins is genuinely less pronounced than the pervasive effect of chronic inflammation driven by bacterial surface antigens like OMPs. Therefore, while this study prioritizes the anti-OMP response as the most robust causal contributor, the role of CagA and VacA cannot be dismissed. Their potential influence requires re-evaluation with more powerful genetic instruments derived from larger exposure GWAS to overcome current limitations.

The strengths of this study include its robust MR design, the use of large-scale GWAS data, and the application of a comprehensive suite of sensitivity and multivariable analyses, in line with STROBE-MR guidelines. This approach allowed us to minimize confounding and dissect correlated exposures to pinpoint a primary causal contributor. However, this study has several limitations. First, our analyses were restricted to individuals of European ancestry to avoid confounding from population stratification. Consequently, the generalizability of these findings to other populations, particularly those in East Asia with a higher burden of GC and different *H. pylori* strain prevalence requires further investigation in dedicated, ancestrally diverse cohorts. Second, while MR is a powerful tool for causal inference, the potential for unmeasured horizontal pleiotropy cannot be completely ruled out, though the sensitivity tests provided no evidence for such bias. Finally, the mediation finding for TNFSF18 should be interpreted with caution given its marginal statistical significance and should be considered hypothesis-generating.

## Conclusions

This MR study provides strong genetic evidence, suggesting that the host immune response to bacterial outer membrane proteins is a key contributor to the causal pathway from *H. pylori* infection to GC. This finding challenges the long-standing focus on specific translocated toxins like CagA and highlights the chronic host-bacterium interface as the critical battleground where oncogenic risk is determined. The potential mediating role of the TNFSF18 pathway offers a new avenue for mechanistic research. Ultimately, these findings could inform more precise risk stratification and prevention strategies that target the specific inflammatory consequences of the host’s immune response to the bacterial surface.
